# HBsAb and HBcAb Have No Significant Effect on the Progression of SLE and RA

**Published:** 2017-08

**Authors:** Fen WANG, Shujuan LV, Jingyu CAI, Xianglian ZHOU, Qicui ZHU, Jianhua XU

**Affiliations:** 1. Dept. of Rheumatology, The First Affiliated Hospital of Anhui Medical University, Hefei, China; 2. Dept. of Microbiology, School of Basic Medical Sciences, Anhui Medical University, Hefei, China

## Dear Editor-in-Chief

Systemic lupus erythematosus (SLE) and rheumatoid arthritis (RA) are common autoimmune diseases, and the global prevalence of chronic hepatitis B virus (HBV) infection is high. The aim of this study was to investigate HBV infection in SLE and RA patients.

This study was conducted at the First Affiliated Hospital of Anhui Medical University in China, 2015. All SLE and RA inpatients were examined for HBV infection. Healthy controls were these who underwent an annual physical examination. This study was approved by the hospital’s ethics committee.

The data were collected using Epi Data 3.1, and the statistical analysis was performed using SPSS version 16.0 (Chicago, IL, USA). Analysis was performed using the χ^2^ test or student’s t-test. Statistical significance was defined as *P-*value<0.05.

The study included 346 SLE patients, 27 males, and 319 females. The average age 37.3±14.0 yr and the mean age of onset were approximately 31.8±13.3 yr. Overall, 294 RA patients were analyzed-55 males and 239 females with an average age of 55.2±12.8 yr and mean onset age of approximately 45.8±14.4 yr. The HBsAg-positive rate in SLE patients was significantly lower than in the healthy controls (0.6% vs. 6.6%, respectively), but in RA patients it was higher (7.1% vs. 6.6%). Accordingly, there was a significant difference in the HBsAg-positive rate between the SLE and RA patients (0.6% vs. 7.1%, respectively; *P*<0.001). Similar SLE-vs.-RA relationships were observed for the positive rates of HBsAb (41.3% vs. 57.8%, *P*<0.001), HBeAb (1.4% vs. 6.8%, *P*<0.001) and HBcAb (2.9% vs.16.3%, *P*<0.001). Next, we investigated the relationship between presence of HBcAb or HBsAb and the clinical following parameters: gender; liver B-ultrasonic and spleen B-ultrasonic findings; Jaundice; levels of ALT, GGT, CRP, RF, CCP, C3, and DsDNA; and disease activity. There was no significant difference between the presence of HBsAb and any of seven clinical parameters between the SLE and RA groups (*P*>0.05 for all). Additionally, no significant differences were found between the presence of HBcAb and the clinical data (*P*>0.05 for all).

In this study, the healthy controls were approximately 12779 people. The prevalence of HBsAg was 6.6%, which was slightly lower than the reported proportion of HBsAg-positive patients in the general Chinese population (1). The lower HBV infection rate is probably due to the nationwide HBV vaccination program started in recent years. The proportion of females among the SLE patients (92.2%) was higher than that among the RA patients (81.3%). The mean age of onset of SLE and RA was 31.8±13.3 and 45.8±14.4 yr, respectively. This finding indicates that young female SLE patients are common in China, and the most RA patients are middle-aged to elderly females. HBV infection in patients with SLE was not frequently observed. The positive rate of HBsAg, indicating HBV infection, was significantly lower in patients with SLE (0.6%) than that in the healthy controls (6.6%). The HBsAg-positive rate was significantly higher in the patients with RA (7.1%) than in those with SLE (0.6%) and the healthy control (6.6%). The most remarkable findings were the low HBeAb- and HBcAb-positive rate in the patients with SLE compared with patients with RA. This finding may suggest a protective role of HBV in SLE as compared with RA. In the current study, the clinical parameters in SLE and RA patients were not associated with the presence of HBsAb and HBcAb. The presence of HBsAb may be the consequence of hepatitis B immunity instead of hepatitis B infection, and the presence of HBcAb, a seromarker of previous or current infection with HBV, may distinguish between infected and vaccinated populations, which include people, infected with or are now contracting HBV. Our preliminary findings suggest the absence of an association between hepatitis B vaccination or chronic HBV infection and levels of ANA, CRP, RF, CCP, DsDNA, C3, and disease activity in SLE and RA patients. Several researchers have proposed that the pathogenesis of HBV-associated arthritis is attributable to the deposition of immune complexes in synovial tissues (2, 3). However, one study in RA patients found no association between chronic HBV infection and disease activity, synovitis, or joint destruction (4). In our study, the effects of hepatitis B immunity and past or current HBV infection on the clinical course of SLE and RA, if any, were relatively minor.

We found an extremely low prevalence of HBsAg in SLE patients and a high prevalence of HBsAg in RA patients compared with healthy controls. Additionally, there was no absence of association between hepatitis B vaccination and chronic HBV infection and disease activity in either the SLE or RA patients.
